# Enzymatic β-Mannosylation of Phenylethanoid Alcohols

**DOI:** 10.3390/molecules30020414

**Published:** 2025-01-19

**Authors:** Lucia Černáková, Peter Haluz, Vladimír Mastihuba, Zuzana Košťálová, Elena Karnišová Potocká, Mária Mastihubová

**Affiliations:** Institute of Chemistry, Slovak Academy of Sciences, Dúbravská Cesta 9, SK-845 38 Bratislava, Slovakia; lucia.cernakova@savba.sk (L.Č.); peter.haluz@savba.sk (P.H.); vladimir.mastihuba@savba.sk (V.M.); zuzana.kostalova@savba.sk (Z.K.);

**Keywords:** phenylethanoid glycosides, β-mannosidase, Novozym 188, tyrosol β-D-mannopyranoside, hydroxytyrosol β-D-mannopyranoside, mannobiose

## Abstract

Phenylethanoid glycosides (PhGs) are widely occurring secondary metabolites of medicinal plants with interesting biological activities such as antioxidant, anti-inflammatory, neuroprotective, antiviral, hepatoprotective, immunomodulatory, etc. They are characterized by a structural core formed by a phenethyl alcohol, usually tyrosol or hydroxytyrosol, attached to β-D-glucopyranose via a glycosidic bond. This core is usually further decorated by attached phenolic acids or another saccharide. Several studies suggest an important role of the saccharidic fragment in the biological activities of PhGs, provoking demand for new glycovariants of natural PhGs. This study presents the preparation of β-mannosylated analogs of tyrosol β-D-glucopyranoside (salidroside) and hydroxytyrosol β-D-glucopyranoside (hydroxysalidroside). While the chemical synthesis of β-D-mannopyranosides is rather challenging, they can be prepared by enzymatic catalysis. We found that Novozym 188, an industrial β-glucosidase, also contains β-mannosidase and used this enzyme in the preparation of tyrosol β-D-mannopyranoside and hydroxytyrosol β-D-mannopyranoside in 12 and 16% chemical yields, respectively, by transglycosylation from β-D-mannopyranosyl-(1→4)-D-mannose. The mannosylation was chemoselective and occurred exclusively on the primary hydroxyls of tyrosol and hydroxytyrosol, and the glycosylation of phenolic moieties of the aglycons was observed.

## 1. Introduction

Plants produce a large number of different secondary metabolites, a large proportion of which are represented by various phenolic compounds. Among them, phenylethanoid glycosides (PhGs), glycosylated natural derivatives of phenylethanoid alcohols such as tyrosol (**1**) or hydroxytyrosol (**2**), possess a wide variety of interesting bioactivities, including antioxidant, anti-inflammatory, neuroprotective, antivirus, immunomodulatory, cytotoxic, etc. [1,2,3]. The simplest PhGs, tyrosol β-D-glucopyranoside (salidroside) and hydroxytyrosol β-D-glucopyranoside (hydroxysalidroside), represent the most frequent core structures of more complex PhGs. These structures are further decorated by attached sugars such as apiose, rhamnose, galactose and phenolic (mostly hydroxycinnamic) acids [3].

In general, the beneficial properties of medicinally relevant glycosides result from the combination of their structural fragments—the glycon part increases the solubility of the bioactive aglycon, thus influencing the pharmacokinetic properties of the whole molecule. On the other hand, sugars in the glycon may be key components for various biorecognition processes, thus enabling or blocking glycoside transport through membranes or its association with various organelles [4,5]. Variation in the sugar moiety can therefore significantly affect the resulting biological activity of the glycoside, including PhGs. Various enzymatic and chemical procedures were therefore developed to prepare non-natural glycovariants of salidroside and hydroxysalidroside to modulate their bioactivities [6,7,8,9,10,11,12,13,14,15]. Effective β-mannopyranosylation of **1** and **2** would help to widen the library of such non-natural PhGs.

**Scheme 1:**
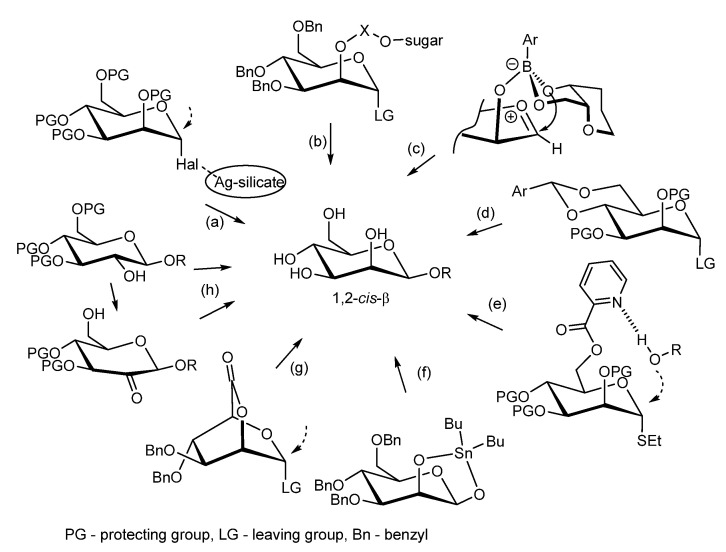
Overview of chemical methods for the preparation of β-D-mannopyranosides. Letters (**a**–**h**) refer to the synthetic strategies of preparation of β-mannopyranosides discussed in the text. Dashed arrows depict the direction of the nucleophile attack.

Stereoselective β-mannopyranosylation is considered one of the most challenging problems in glycochemistry for two reasons. First, the anomeric effect disfavors the formation of the β-linkage and favors the formation of the preferred α-mannopyranoside in the 1,2-*trans* configuration. Second, the 1,2-*cis* configuration of the equatorial aglycone and the axial functionality at C-2 in β-mannopyranosides have repulsive steric effects. In recent years, various strategies have been developed to solve this problem [16,17,18,19], such as (a) the Ag-silicate-promoted S_N_2 glycosidation of *α*-mannosyl halides [20], (b) intramolecular aglycon delivery (IAD) [21,22], (c) boron-mediated IAD with 1,2-anhydrosugars [23,24] (d) the Crich method employing 4,6-benzyliden triflate generated in situ [25,26,27] and other 4,6-tethered strategies [28], (e) hydrogen-bonding aglycone delivery (HAD) [29,30], (f) the stannylene acetal method [31], (g) the S_N_2-type reaction using a 2,6-lactone-bridged donor [32,33,34] and other indirect approaches such as (h) C-2 inversion [35] or C-2 oxidation–reduction [36] (Scheme 1). Additional combined or novel approaches to β-mannosylation are constantly being published [37,38,39,40]. All mentioned methods, however, may suffer either from the risk of the formation of both product anomers or from requiring a series of several synthetic steps, oftentimes under sensitive reaction conditions.

The lack of efficient methods for stereoselective β-mannosylation has provoked the development of broadly applicable one-pot enzymatic glycosylation strategies. Due to selectivity issues, tailored de novo synthesis of β-mannosylated glycans is preferably carried out under the catalysis of specific enzymes such as mannosyl transferases and mannosyl phosphorylases [41,42]. The scale of these reactions is, however, limited by the availability and cost of enzymes and β-mannosyl donors. On the other hand, the β-mannosylation of aliphatic alcohols [43,44,45,46,47] or mono-glycosides [48] can be catalyzed by β-mannosidases as relatively abundant and inexpensive glycosyl hydrolases. The use of β-mannosidases in the synthesis of alkyl β-mannosides also has some limitations: probably due to the mentioned steric effects, direct mannosylation by reversed hydrolysis is ineffective, and transglycosylation is the preferred way of preparing the target β-mannosides. Moreover, reversed hydrolysis does not allow the use of inexpensive non-purified industrial enzymes as catalysts, because the potential occurrence of α-mannosidase side-activity in the crude enzyme preparation could catalyze collateral α-mannosylation.

The prices of substrates serving as donors for transglycosylations catalyzed by β-mannosidases significantly influence the cost of the transmannosylation—while the price of 4-nitrophenyl β-D-mannopyranoside makes this synthetic substrate impractical for preparative reactions at a larger scale, the price of mannobiose (β-D-mannopyranosyl-(1→4)-D-mannose) varies depending on the supplier and purity, since this substrate can be produced by the hydrolysis of natural polysaccharides such as mannans, galactomannans, glucomannans, etc.

Within our research on the application of glycosidases in preparative glycosylations, we found that the industrial β-glucosidase Novozym 188 comprises a substantial level of β-mannosidase side-activity, and we used this enzyme to catalyze the synthesis of tyrosol and hydroxytyrosol β-D-mannopyranosides using mannobiose as the β-mannosyl donor.

## 2. Results and Discussion

**Figure 1:**
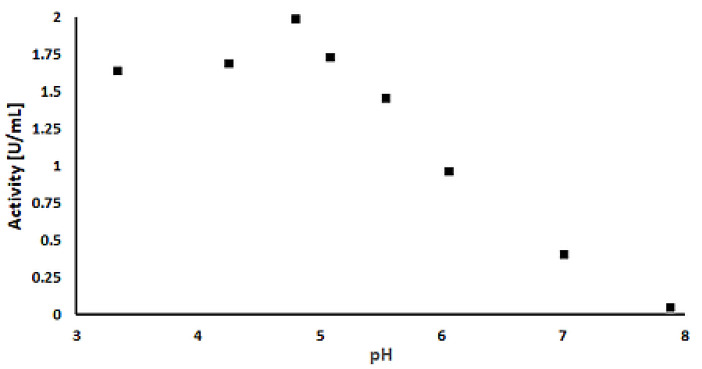
Effect of pH on β-mannosidase activity in Novozyme 188.

The goal of this work was to present a new, simple and inexpensive method of the synthesis of β-D-mannopyranosides and to demonstrate it on the β-mannosylation of tyrosol **1** and hydroxytyrosol **2** to obtain new glycovariants of natural phenylethanoid glycosides. The reaction was carried out as transglycosylation catalyzed by Novozyme 188. Novozyme 188 is a crude secretome of *Aspergillus niger* sold as an industrial β-glucosidase. We have found that in addition to other glycosidase activities, this crude enzyme mixture also contains a significant level of β-mannosidase (Table 1). The mannosidase is more active in acidic media, and its activity has an optimum close to pH 5 (Figure 1).

**Scheme 2:**
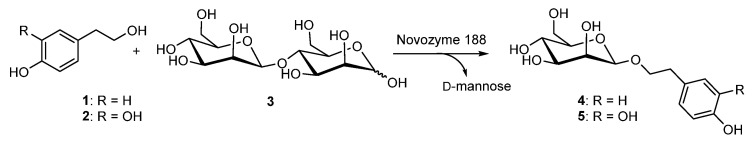
Mannosylation of tyrosol **1** and hydroxytyrosol **2** by β-mannosidase in Novozym 188.

In preparative reactions, the ultrafiltered and lyophilized form of Novozyme 188 was used to increase the β-mannosidase activity in the reaction mixtures and to avoid the presence of impurities and free sugars from the crude enzyme. The enzyme lyophilisate used in this work comprised 22.6 U/g β-mannosidase, which was sufficient to catalyze the demanded mannosylations of tyrosol and hydroxytyrosol. Mannobiose (**3**) was used as the β-mannosyl donor (Scheme 2). Since other saccharides were not present in the reaction mixture, the side-activities of glycosidases occurring in Novozym 188 did not influence the reaction.

The ^1^H NMR spectra of the produced tyrosol β-D-mannopyranoside **4** and tyrosol β-D-mannopyranoside **5** show only one signal for an anomeric proton (δ 4.47 ppm for both compounds). The anomeric configuration of **4** and **5** was determined through the directly bonded ^13^C1−^1^H1 coupling constant (**4**: ^1^*J*_C1−H1_ = 156.4 Hz; **5**: ^1^*J*_C1−H1_ = 157.2 Hz) [49] (Supplementary Materials, Figures S2 and S4). The chemical shift in the proton H-5 of mannosides **4** and **5** was 3.18 ppm for both compounds. In both cases, the phenethyl moiety was linked to the mannose via the primary hydroxyl. The methylene group closer to the newly formed glycosidic bond CH_2_α showed separated multiplet signals for protons (δ 4.05 (dt, *J* = 9.7, 7.0 Hz, 1H, CH_2_αa); 3.75–3.66 (m, 2H, CH_2_αb, H-6b) for **4** and 4.04 (dt, *J* = 9.8, 7.1 Hz, 1H, CH_2_αa); 3.75–3.66 (m, 2H, CH_2_αb, H-6b) for **5**). The methylene group CH_2_β attached to phenyl displayed one multiplet signal (δ 2.81 (t, *J* = 7.2 Hz, 2H, CH_2_β) for **4** and 2.76 (t, *J* = 7.2 Hz, 2H, CH_2_β) for **5**) (Supplementary Materials, Figures S1 and S3). This means that although several glycosidase-catalyzed glycosylations of the phenolic moiety of tyrosol have been recently reported [6,7,50,51], the β-mannosylations of **1** and **2** catalyzed by Novozym 188 proceeded exclusively on the primary hydroxyls of aglycones, and no products of phenol glycosylation were observed in the reaction mixtures (Scheme 2).

**Figure 2:**
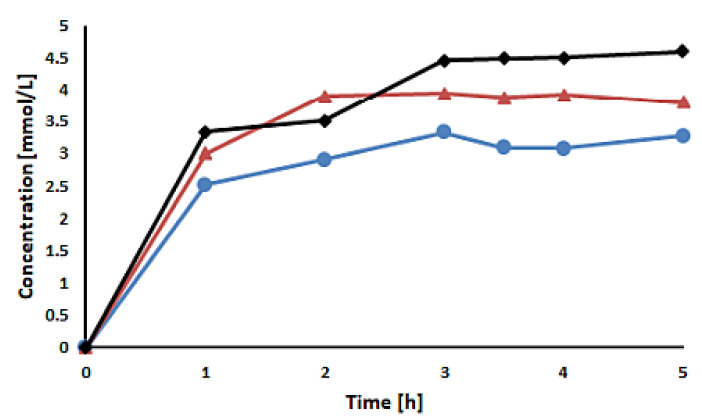
Time course of production of tyrosol β-D-mannopyranoside catalyzed by Novozym 188—effect of tyrosol concentration. Concentration of mannobiose—30 g/L; activity of β-mannosidase—90.4 U/L. Concentrations of tyrosol: 10 g/L (-●-), 15 g/L (-▲-) and 20 g/L (-♦-) in 0.1 mol/L acetate buffer pH 5.

To establish the conditions for preparative β-mannosylations, the reaction was first studied at a small scale to assess the influence of the concentration of the glycosyl donor **3** and the acceptor **1**. Three concentrations of **1** were tested to evaluate the effect of the acceptor on the formation of **4**. In contrast to reversed hydrolysis, transglycosylations are usually carried out with low concentrations of the glycosyl donor. The initial concentration of **3** was therefore set at 30 g/L, corresponding to 88 mmol/L. Increasing the concentration of tyrosol up to 20 g/L, which is close to its solubility in water, resulted in increased production of the tyrosol β-D-mannopyranoside **4** (Figure 2).

Transglycosylation is a kinetically controlled process during which the concentration of the glycosylated product reaches a peak, after which its concentration decreases due to product secondary hydrolysis, which outweighs its formation since a substantial part of the glycosyl donor is consumed. The reaction must therefore be monitored and stopped at the right time to avoid product losses. A suitable initial concentration of the glycosyl donor has to be found to ensure sufficient product formation. The low production of **4** in previous experiments suggests that higher concentrations of mannose donor **3** and longer reaction times are required to achieve better productivity in the mannosylation of **1**.

**Figure 3:**
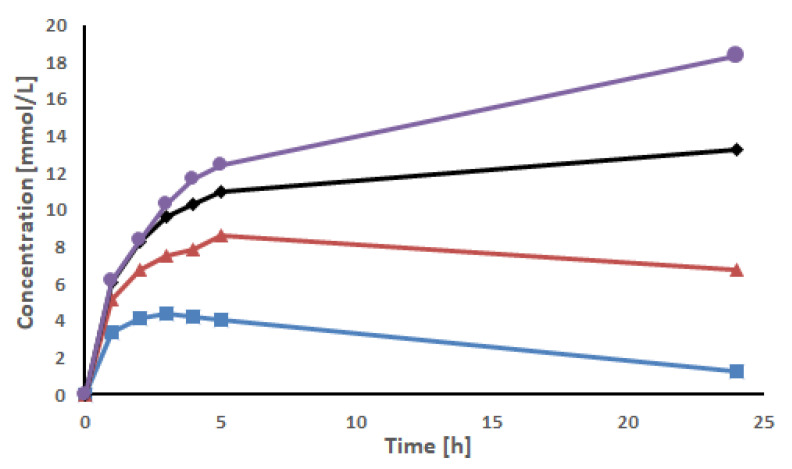
Time course of production of tyrosol β-D-mannopyranoside catalyzed by Novozym 188—effect of mannobiose concentration. Concentration of tyrosol—20 g/L; activity of β-mannosidase—90.4 U/L. Concentrations of mannobiose: 30 g/L (-■-), 60 g/L (-▲-), 90 g/L (-♦-) and 120 g/L (-●-) in 0.1 mol/l acetate buffer pH 5.

The highest concentration of **1** studied (20 g/L) was chosen in experiments optimizing the starting concentration of **3**. At low concentrations of **3** (30 and 60 g/L), the point when secondary hydrolysis dominates over its production was reached within 5 h, while the production of **4** increased continuously in reactions with **3** starting concentrations of 90 and 120 g/L (Figure 3). The conversions of **3** to **4** after 24 h were almost identical (5.1 and 5.2%, respectively) in the latter two reactions, suggesting that an increase in the starting concentration of costly mannobiose above 120 g/L is not necessary. Under such conditions, 6.1 mmol/L of **4** was produced within one hour in the reaction mixture containing 4 g/L of the lyophilized preparation of Novozym 188. This corresponds to the transglycosylation activity of β-mannosidase, at 25.4 U/g, which is comparable to the hydrolytic activity, at 22.6 U/g.

**Figure 4:**
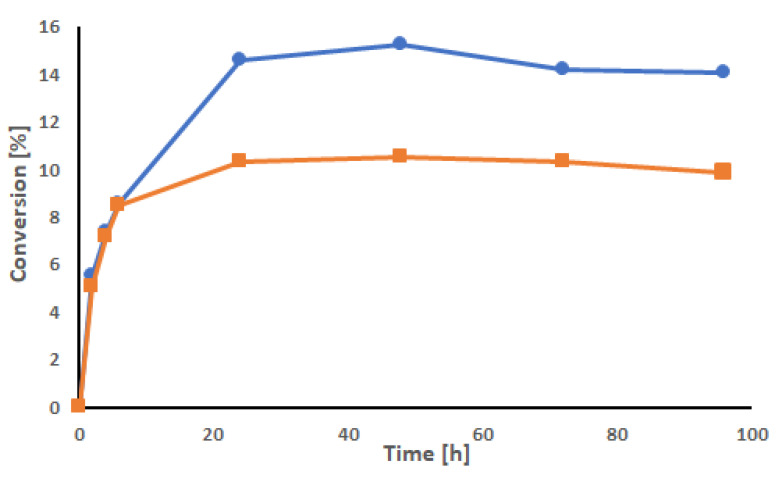
Time course of conversion of tyrosol to tyrosol β-D-mannopyranoside (-■-) and hydroxytyrosol to hydroxytyrosol β-D-mannopyranoside (-●-) catalyzed by Novozym 188. Concentration of mannobiose—120 g/L; activity of β-mannosidase—90.4 U/L. Concentration of tyrosol and hydroxytyrosol—20 g/L in 0.1 mol/L acetate buffer pH 5.

Based on the previous results, the conditions for the transglycosylations of **1** and **2** were selected to be 20 g/L of aglycone and 120 g/L of **3**. In order to find the optimum reaction times, long-term experiments (96 h) were carried out. In both reactions, secondary product hydrolysis occurred only to a limited extent, again suggesting a sufficient amount of the mannosyl donor present in the reaction mixtures. The conversion of aglycones to mannosylated products slightly peaked at 48 h, although the reactions can be stopped at any time between 24 and 96 h without a substantial loss of the product (Figure 4). A longer reaction time of hydroxytyrosol mannosylation should, however, be avoided due to the production of colored side-products, which does not occur in the mannosylation of tyrosol. Surprisingly, hydroxytyrosol **2** was mannosylated to a higher degree, with the maximum concentration of **5** in the reaction mixture reaching 19.8 mmol/L, corresponding to a conversion of **2** to **5** of 15.3%, contrary to the maximum concentration of **4** of 15.2 mmol/L, corresponding to a conversion of 10.5% relative to **1**.

The preparative reactions were executed under optimized conditions at a 25 mL scale. Reactions stopped after 48 h provided **4** and **5** in 12 and 16% chemical yields, respectively. The yields are slightly higher than the maximum conversions found during the optimization, probably due to better material transfer during stirred batch reactions compared to during the optimization reactions in an analytical scale shaken on a thermoshaker. Glycosidase-catalyzed β-mannosylations published so far report the synthesis of alkyl β-D-mannopyranosides in yields ranging from 2 to 80%, depending on the used mannosyl donor (various mannooligosaccharides and 4-nitrophenyl β-D-mannopyranoside), the chemical nature of the aglycone and the source of the enzyme. The best results were achieved with short aliphatic alcohols well miscible with water, with secondary alcohols also being glycosylated to a limited extent [43,44,45,46,47]. To the best of our knowledge, no β-mannosylations of phenylethanoid alcohols have been reported.

## 3. Materials and Methods

### 3.1. Enzymes and Chemicals

Novozym 188 (*Aspergillus niger*) was obtained from Novozymes (Bagsvaerd, Denmark). The enzyme was ultrafiltered and lyophilized before use in transglycosylations. Tyrosol (97%) was purchased from Maybridge (Loughborough, UK), chloroform from Centralchem (Bratislava, Slovakia) and HPLC-grade methanol from Merck KGaA (Darmstadt, Germany). Celite® 545 was a product of Alfa Aesar (Karlsruhe, Germany), Diaion® HP-20 was a product of Supelco (Bellefonte, PA, USA) and KP-Sil SNAP cartridge from Biotage (Uppsala, Sweden). Hydroxytyrosol was prepared according to Zhang et al. [52]. Mannobiose **3** was a product of the Institute of Chemistry, Slovak Academy of Sciences (Bratislava, Slovakia). 4-nitrophenyl α-D-glucopyranoside, 4-nitrophenyl β-D-glucopyranoside, 4-nitrophenyl α-D-galactopyranoside, 4-nitrophenyl β-D-galactopyranoside, 4-nitrophenyl α-L-arabinopyranoside, 4-nitrophenyl α-L-arabinofuranoside, 4-nitrophenyl α-L-rhamnopyranoside, 4-nitrophenyl β-D-mannopyranoside and 4-nitrophenyl β-D-xylopyranoside were products of Biosynth (Bratislava, Slovakia). The β-D-mannopyranosides of tyrosol and hydroxytyrosol used as standards for HPLC were prepared by non-optimized enzymatic transglycosylations. Silica gel 60 for flash chromatography was obtained from Fluka (Buchs, Switzerland). TLC was performed on alumina plates with Silica gel 60 F254 from Merck KGaA (Darmstadt, Germany).

### 3.2. Apparatus

High-performance liquid chromatography was performed on an Agilent 1200 Series apparatus (Agilent Technologies, Inc., Santa Clara, CA, USA) consisting of a quaternary pump, RI and UV–VIS detectors, a column thermostat and a Rheodyne injector with a 20 µL loop. Flash chromatography was performed on an Isolera One from Biotage (Uppsala, Sweden) using 50 g KP-Sil SNAP cartridges. The structures of the products were determined by a combination of ^1^H and ^13^C NMR spectroscopy as well as two-dimensional homonuclear and heteronuclear techniques (COSY, HSQC), and recorded on a 400 MHz Bruker AVANCE III HD (Bruker GmbH, Karlsruhe, Germany) equipped with a Prodigy CryoProbe (Bruker GmbH, Karlsruhe, Germany).

### 3.3. Enzymatic Reactions

#### 3.3.1. Measurements of Glycosidase Activities in Novozyme 188

Assay of glycosidase activities: A portion of properly diluted enzyme was mixed with the respective nitrophenyl glycoside diluted in 0.1 M acetate buffer with a pH of 5 (final concentration of 1 mM) and incubated at 37 °C and 500 rpm. Portions of 250 µL were withdrawn after 5, 10 and 15 min, mixed with 1.25 mL of a saturated solution of borax and filtered, and the formation of free nitrophenol was measured at 410 nm. One glycosidase unit corresponds to the amount of enzyme which catalyzes hydrolysis for 1 µmol of substrate in one minute. Activity was calculated from data obtained after 5 min of reaction; measurements after 10 and 15 min served as a control of the linearity of the reaction.

The optimal pH of β-mannosidase activity was tested on 1 mM 4-nitrophenyl β-D-mannopyranoside in a 0.1 McIlvain buffer of varying pH.

#### 3.3.2. Selection of Reaction Conditions for Enzymatic β-Mannosylations of Tyrosol

The mannosylation of tyrosol **1** was optimized according to the concentration of mannobiose **3** as a glycosyl donor and the concentration of aglycone **1** in 0.1 M acetate buffer with pH 5.0 in 1.5 mL Eppendorf tubes at 500 rpm and 37 °C. To investigate the synthetic process, 100 µL aliquots were withdrawn in predefined time intervals, mixed with 3 volumes of methanol to quench the reaction and filtered through a 0.22 µm nylon syringe filter. A 100 µL portion of the filtrate was mixed with 4 volumes of distilled water and used for analysis by HPLC. The concentrations of reactants were calculated as averages from three parallel reactions. The concentration of **1** was tested in a range of 72 to 145 mmol/L, and the concentration of **3** ranged from 88 to 351 mmol/L. The activity of β-mannosidase in all enzymatic reactions was 90.4 U/L.

The time course of the β-mannosylation of **2** at a 1 mL scale was monitored in 3 parallel reactions under the same reactant concentrations as those selected for the preparative β-mannosylation of 1. Conversions to β-mannopyranosides were expressed as percentage ratios of their molar mass toward starting reactants according to the discussed context.

Examples of chromatograms of reaction mixtures are available in Supplementary Materials Figures S5 and S6.

#### 3.3.3. Preparation of **4** and **5**

Aglycone **1** or **2** (0.5 g) and **3** (3 g) were dissolved in 20 mL of 0.1 M acetate buffer with pH 5 and pre-tempered at 37 °C. A total of 5 mL of Novozym 188 dissolved in the same buffer (2.26 U) was added, and the reaction mixture was stirred for 48 h at 37 °C and then stopped by boiling in a microwave oven, cooled down and filtered. The filtrate was applied to a 50 g KP-Sil SNAP cartridge filled with Diaion^®^ HP-20 and flushed with water until all free sugars were eluted out. Then, the mannosylated product and free aglycone were together eluted by ethanol. Celite^®^ 545 was added to the ethanolic fraction which was then evaporated to dryness on a rotary evaporator. Dry powder was applied on the top of the silica gel column equilibrated in chloroform. Aglycone and its respective mannosylated product were separated by flash chromatography with a gradient of methanol in chloroform.

Fractions containing the pure product were pooled, and the organic solvents were evaporated to produce either 130 mg (12%) of **4** or 164 mg (16%) of **5**.

### 3.4. Structural Characteristics of Products

#### 3.4.1. Tyrosol β-D-Mannopyranoside (**4**)

White amorphous solid; [α]_D_^20^ = −70.7° (*c* = 1.0, CH_3_OH).

^1^H NMR (400 MHz, CD_3_OD) δ 7.06 (d, *J* = 8.4 Hz, 1H, H-Ph), 6.70 (d, *J* = 8.5 Hz, 1H, H-Ph), 4.47 (s, 1H, H-1), 4.05 (dt, *J* = 9.7, 7.0 Hz, 1H, CH_2_αa), 3.87 (dd, *J* = 11.8, 2.4 Hz, 1H, H-6a), 3.82 (d, *J* = 3.2 Hz, 1H, H-2), 3.75–3.66 (m, 2H, CH_2_αb, H-6b), 3.55 (t, *J* = 9.5 Hz, 1H, H-4), 3.41 (dd, *J* = 9.4, 3.3 Hz, 1H, H-3), 3.18 (ddd, *J* = 9.6, 5.8, 2.4 Hz, 1H, H-5), 2.81 (t, *J* = 7.2 Hz, 2H, CH_2_β).

^13^C NMR (101 MHz, CD_3_OD) δ 156.8 (C-Ph), 130.9 (CH-Ph), 130.8 (C-Ph), 116.1 (CH-Ph), 101.7 (d, ^1^*J_C1H1_* = 156.4 Hz, C-1), 78.3 (C-5), 75.3 (C-3), 72.5 (C-2), 71.8 (CH_2_α), 68.6 (C-4), 62.9 (C-6), 36.3 (CH_2_β).

HRMS (ESI): calcd. for C_14_H_20_O_7_ [M + Na]^+^ = 323.11012, found 323.11017.

#### 3.4.2. Hydroxytyrosol β-D-Mannopyranoside (**5**)

Yellowish foam; [α]_D_^20^ = −60.9° (*c* = 1.0, CH_3_OH).

^1^H NMR (400 MHz, CD_3_OD) δ 6.68 (d, *J* = 2.1 Hz, 1H, H-Ph), 6.67 (d, *J* = 8.1 Hz, 1H, H-Ph), 6.55 (dd, *J* = 8.0, 2.1 Hz, 1H, H-Ph), 4.47 (d, *J* = 0.9 Hz, 1H, H-1), 4.04 (dt, *J* = 9.8, 7.1 Hz, 1H, CH_2_αa), 3.87 (dd, *J* = 11.8, 2.4 Hz, 1H, H-6a), 3.83 (dd, *J* = 3.3, 0.9 Hz, 1H, H-2), 3.75–3.66 (m, 2H, CH_2_αb, H-6b), 3.56 (t, *J* = 9.6 Hz, 1H, H-4), 3.41 (dd, *J* = 9.4, 3.2 Hz, 1H, H-3), 3.18 (ddd, *J* = 9.6, 5.7, 2.4 Hz, 1H, H-5), 2.76 (t, *J* = 7.2 Hz, 2H, CH_2_β).

^13^C NMR (101 MHz, CD_3_OD) δ 146.1 (C-Ph), 144.7 (C-Ph), 131.6 (C-Ph), 121.2 (CH-Ph), 117.1 (CH-Ph), 116.3 (CH-Ph), 101.7 (d, ^1^*J_C1H1_* = 157.2 Hz, C-1), 78.3 (C-5), 75.3 (C-3), 72.5 (C-2), 71.7 (CH_2_α), 68.6 (C-4), 62.8 (C-6), 36.6 (CH_2_β).

HRMS (ESI): calcd. for C_14_H_20_O_8_ [M + Na]^+^ = 339.10504, found 339.10500.

## 4. Conclusions

To prepare new, yet unknown glycovariants of salidroside and hydroxysalidroside, β-D-mannopyranosides of tyrosol and hydroxytyrosol were synthetized by enzymatic transmannosylations from mannobiose in 12 and 16% yields, respectively. The reaction was catalyzed by crude industrial enzyme Novozym 188, comprising β-mannosidase in addition to its main β-glucosidase activity. Both reactions were regioselective, and β-mannosylation occurred exclusively on the primary hydroxyls of tyrosol and hydroxytyrosol. The reaction format described in this report can be used as an alternative to the relatively demanding chemical β-mannosylations of natural products.

## Figures and Tables

**Table 1:** Glycosidase activities in Novozym 188.

Activity	U/mL
α-D-Glucosidase	2.4
β-D-Glucosidase	106.5
α-D-Galactosidase	24.8
β-D-Galactosidase	2.7
α-L-Arabinofuranosidase	8.2
α-L-Arabinopyranosidase	0.11
α-L-Rhamnosidase	1.1
β-D-Mannosidase	1.9
β-D-Xylosidase	0.14

## Data Availability

Data are contained within the article.

## Supplementary Materials

The following supporting information can be downloaded at https://www.mdpi.com/article/10.3390/molecules30020414/s1, Figure S1. ^1^H NMR of 4-hydroxyphenethyl β-D-mannopyranoside (**4**); Figure S2. ^13^C NMR of 4-hydroxyphenethyl β-D-mannopyranoside (**4**); Figure S3. ^1^H NMR of 3,4-dihydroxyphenethyl β -D-mannopyranoside (**5**); Figure S4. ^13^C NMR of 3,4-dihydroxyphenethyl β-D-mannopyranoside (**5**); Figure S5. HPLC chromatogram of reaction mixture from β-mannosylation of tyrosol (**1**) to 4-hydroxyphenethyl β-D-mannopyranoside (**4**) after 48 h; Figure S6. HPLC chromatogram of reaction mixture from β-mannosylation of hydroxytyrosol (**2**) to 3,4-dihydroxyphenethyl β-D-mannopyranoside (**5**) after 48 h.
