# Supplementary material for: Enzymatic β-Mannosylation of Phenylethanoid Alcohols
Source: Molecules. 2025 Jan 19;30(2):414. doi: 10.3390/molecules30020414 (PMC11767590; doi:10.3390/molecules30020414)
Supplement: Supplementary file 1 [file molecules-30-00414-s001.zip › molecules-3405903-supplementary.pdf]

Supplementary Materials

Enzymatic β-Mannosylation of Phenylethanoid Alcohols

Lucia Černáková, Peter Haluz, Vladimír Mastihuba, Zuzana Košťálová, Elena Karnišová Potocká, Mária Mastihubová*

Institute of Chemistry, Slovak Academy of Sciences, Dúbravská Cesta 9, SK-845 38 Bratislava, Slovakia;
lucia.cernakova@savba.sk (L.Č.); peter.haluz@savba.sk (P.H.);
vladimir.mastihuba@savba.sk (V.M.); zuzana.kostalova@savba.sk (Z.K.);
elena.potocka@savba.sk (E.K.P.)

* Correspondence: maria.mastihubova@savba.sk

^{1}H and ^{13}C NMR spectra of phenylethanoid β-mannosides 4 and 5
Figures S1 – S4

HPLC chromatograms of reaction mixtures from β-mannosylation of 1 and 2
Figures S5 – S6

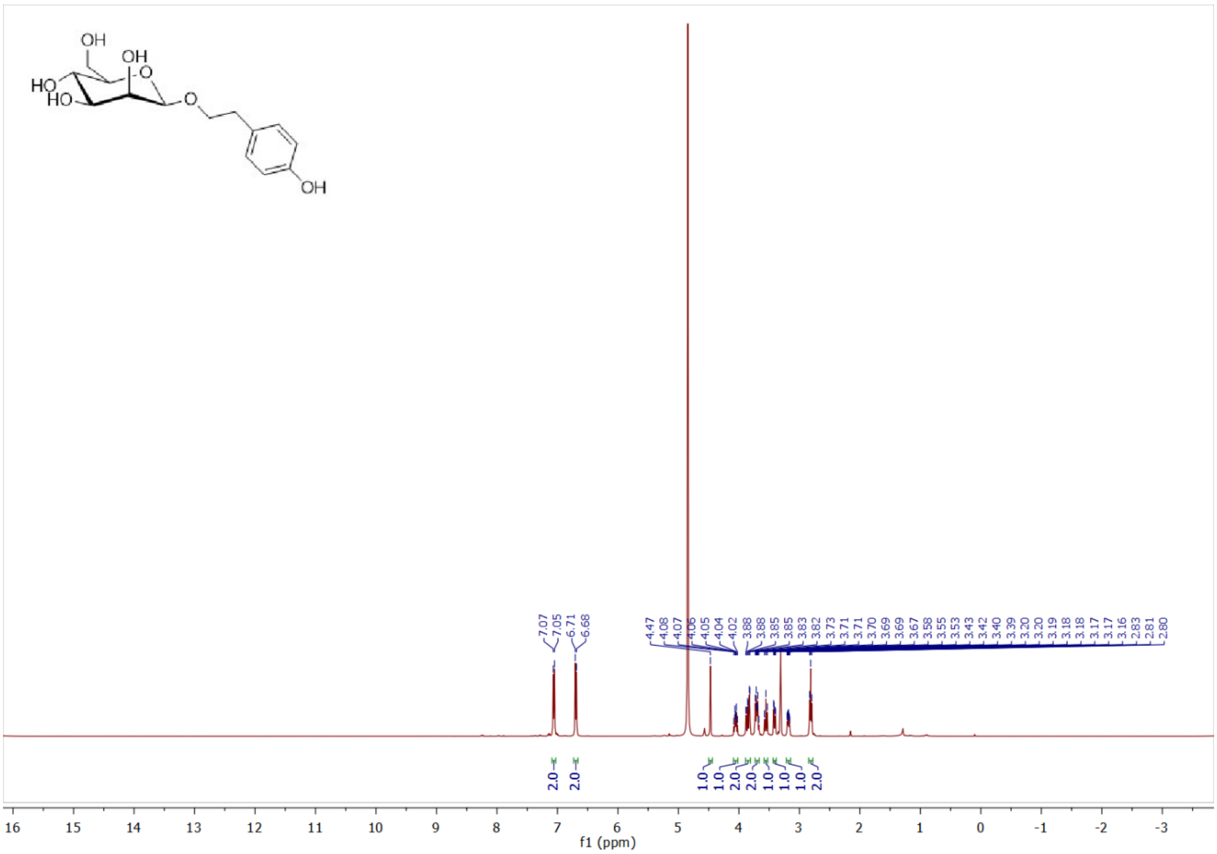

Figure S1. ^{1}H NMR of 4-hydroxyphenethyl β-D-mannopyranoside (**4**)

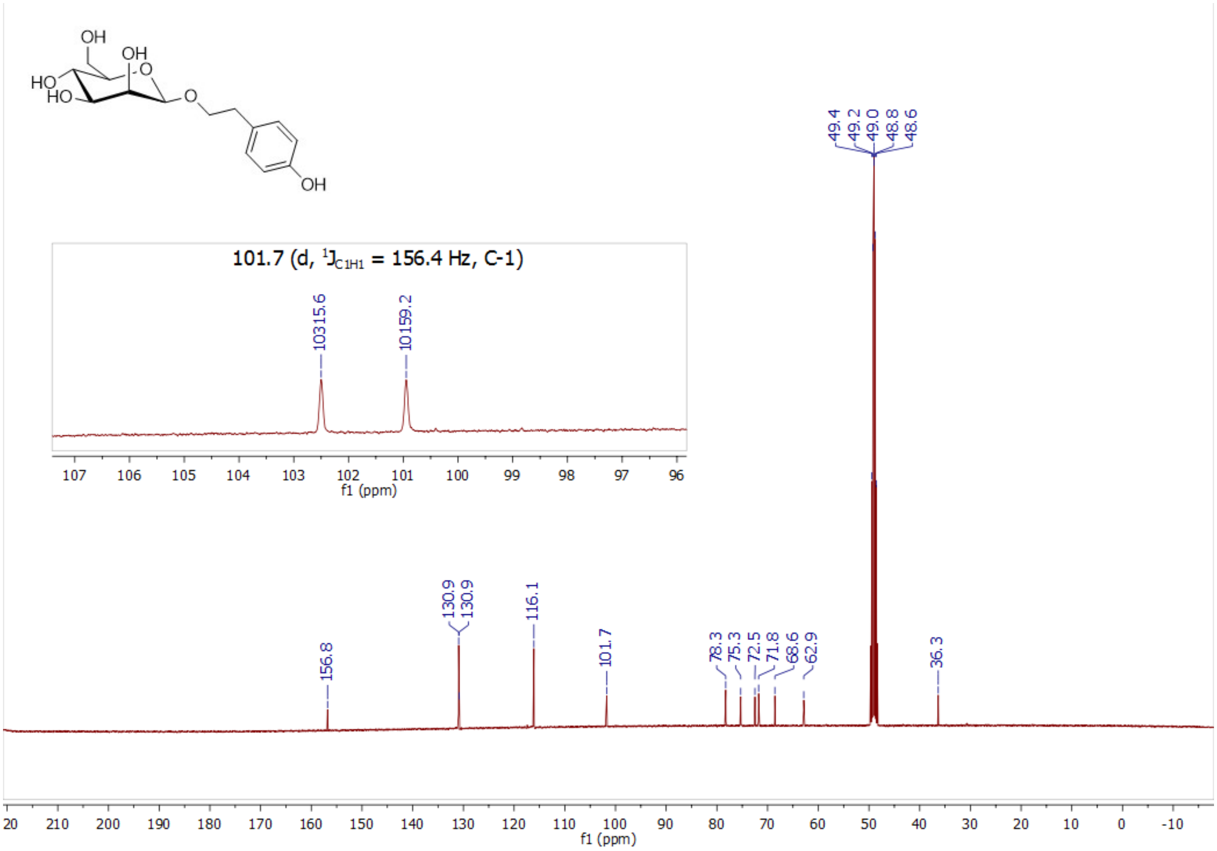

Figure S2. ^{13}C NMR 4-hydroxyphenethyl β-D-mannopyranoside (**4**)

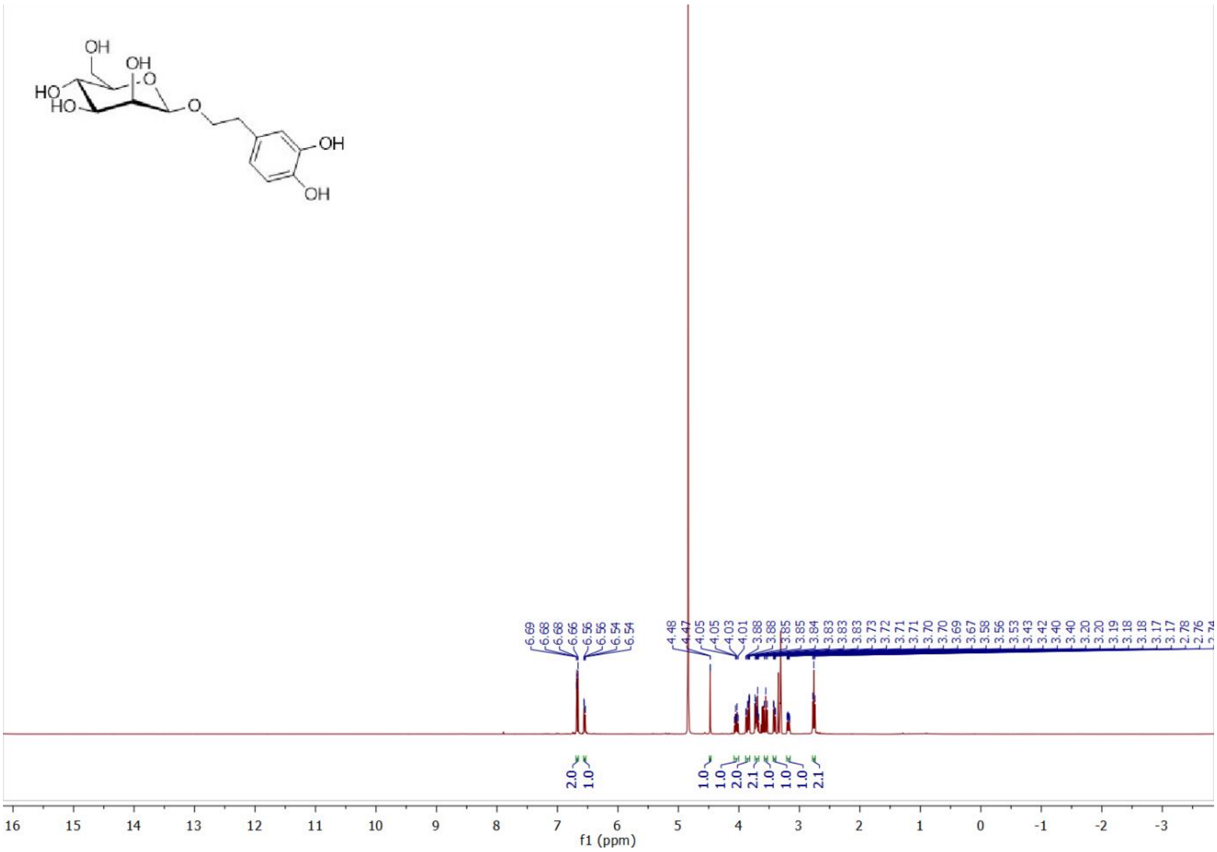

Figure S3. ^{1}H NMR of 3,4-dihydroxyphenethyl β-D-mannopyranoside (**5**)

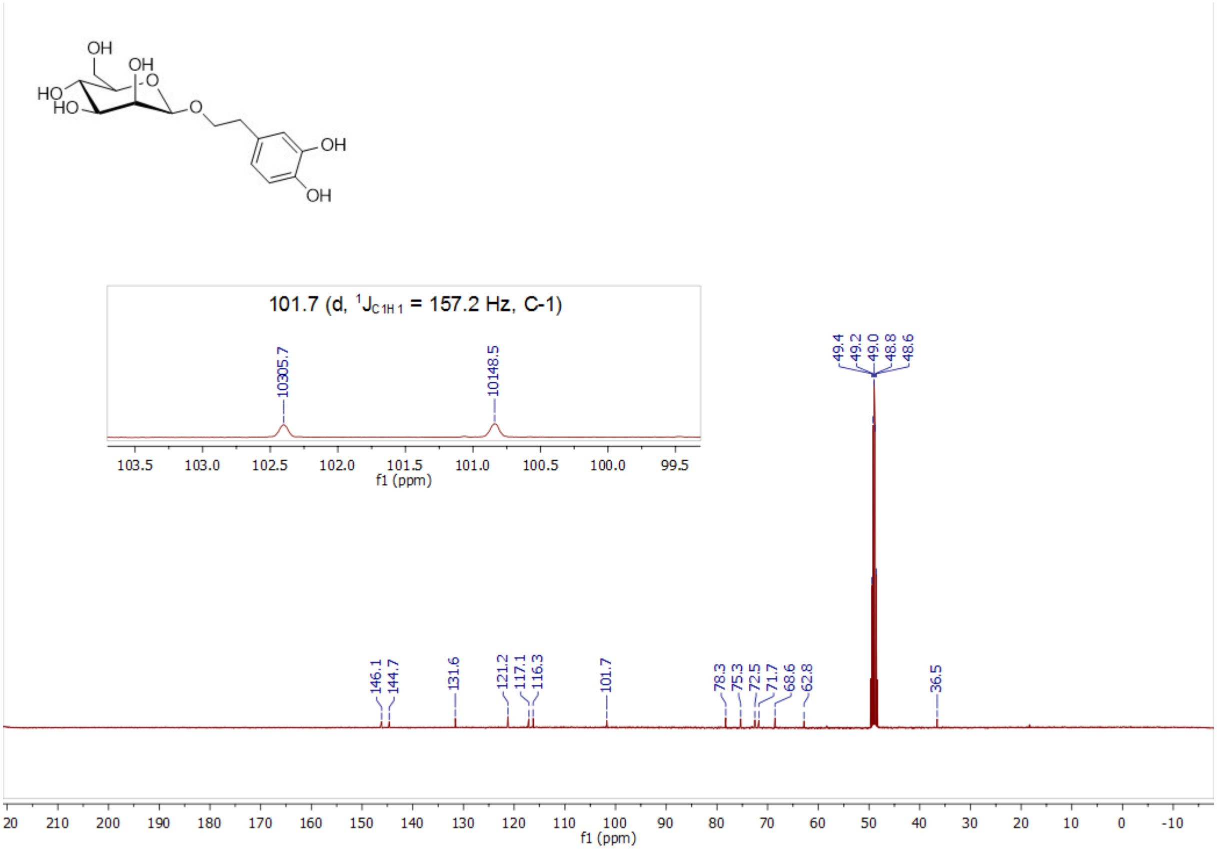

Figure S4. ^{13}C NMR of 3,4-dihydroxyphenethyl β-D-mannopyranoside (**5**)

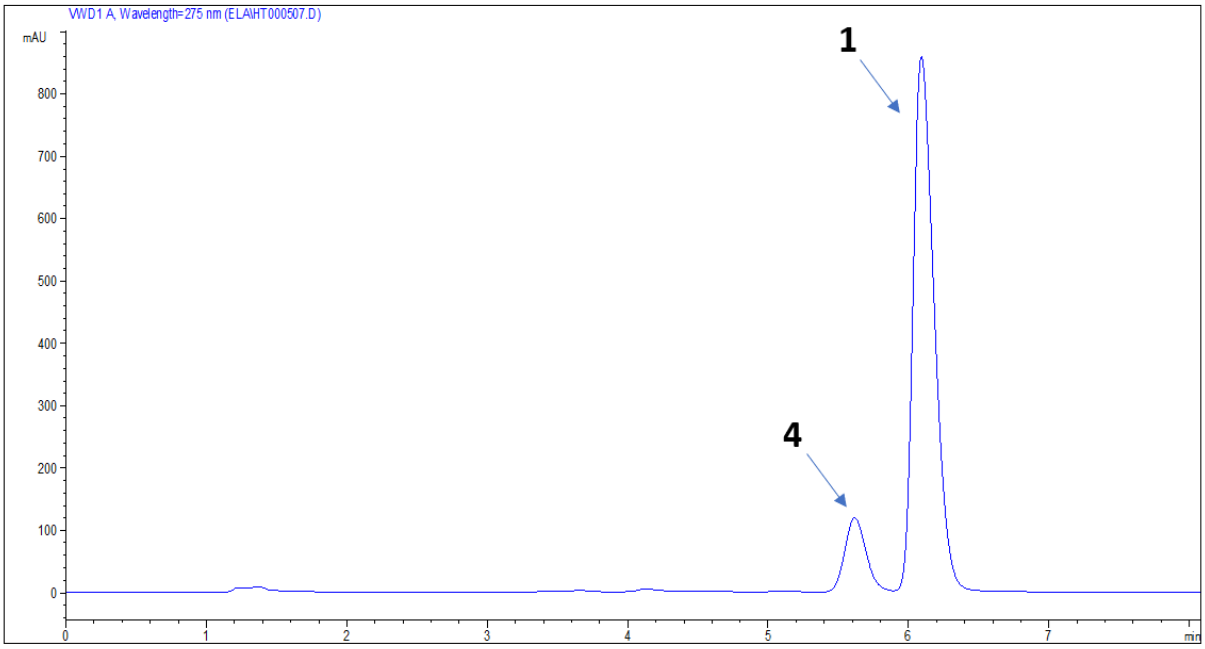

Figure S5. HPLC chromatogram of reaction mixture from β-mannosylation of tyrosol (**1**) **to** 4-hydroxyphenethyl β-D-mannopyranoside (**4**) after 48 h

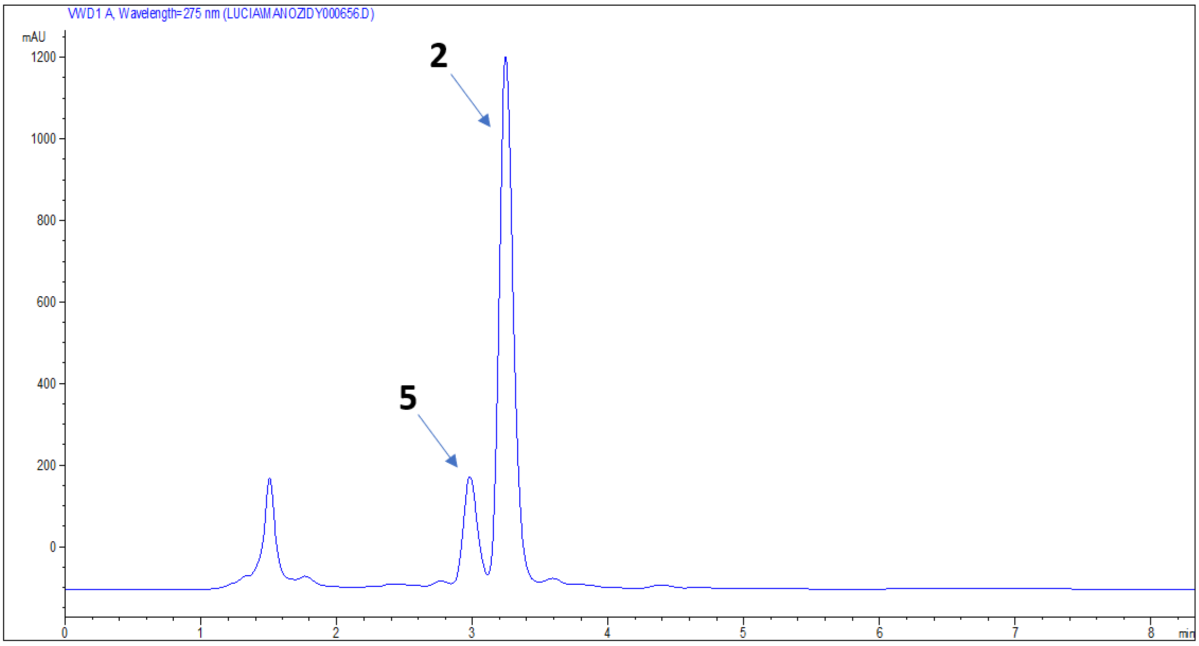

Figure S6. HPLC chromatogram of reaction mixture from β-mannosylation of hydroxytyrosol (**2**) **to** 3,4-dihydroxyphenethyl β-D-mannopyranoside (**5**) after 48 h
